# Lactobacilli and Bifidobacteria: A Parapostbiotic Approach to Study and Explain Their Mutual Bioactive Influence

**DOI:** 10.3390/foods13182966

**Published:** 2024-09-19

**Authors:** Clelia Altieri, Alfonso Filippone, Antonio Bevilacqua, Maria Rosaria Corbo, Milena Sinigaglia

**Affiliations:** 1 Department of Agriculture, Food, Natural Resources and Engineering, University of Foggia, 71122 Foggia, Italy; clelia.altieri@unifg.it (C.A.); alfonso.filippone@unifg.it (A.F.); mariarosaria.corbo@unifg.it (M.R.C.); milena.sinigaglia@unifg.it (M.S.); 2 Department of Psychology and Education, Pegaso University, 80143 Napoli, Italy

**Keywords:** cell-free supernatant, interaction, growth stimulation, protective effect, strain dependence

## Abstract

Three strains of *Lactiplantibacillus plantarum* and three bifidobacteria (*Bifidobacterium animalis* subsp. *lactis*, *Bifidobacterium breve*, and *Bifidobacterium subtile*) were used as target strains; in addition, for each microorganism, the cell-free supernatant (CFS) was produced and used as an ingredient of the growth medium. Namely CFSs from lactobacilli were used on bifidobacteria and CFSs from bifidobacteria were used on lactobacilli. The viable count was assessed, and the data were modelled through a reparametrized Gompertz equation cast both in the positive and negative form to evaluate the parameters t-7log, which is the time after which the viable count was 7 log CFU/mL, and the t-7log*, which is the time after which the viable count was below 7 log CFU/mL; the difference between the t-7log* and t-7log defines the stability time. Statistics through a multiparametric ANOVA (analysis of variance) provided evidence for the presence of a bifidogenic and/or bioactive factor produced by bifidobacteria and active on lactobacilli, and vice versa (bioactive factor of lactobacilli with a functional effect on bifidobacteria), although further studies are required to better explain the mechanisms beyond the positive effects. In addition, the influence on the target strains can be found during the growth phase (stimulation), as well as during senescence and death phase (protective effect), with a strong strain/species dependence on both CFS production and target strain.

## 1. Introduction

Probiotics are “live microorganisms that, when administered in adequate amounts, confer a health benefit on the host” [1]. Probiotics have several health benefits, including the modulation of the gut microbiome; however, techno-functional limitations such as viability controls have hampered their full potential applications in the food and pharmaceutical sectors [2,3].

The actual focus is gradually shifting from viable probiotic bacteria towards non-viable paraprobiotics and/or probiotic-derived biomolecules, so-called postbiotics, defined as a “preparation of inanimate microorganisms and/or their components that confers a health benefit on the host” [4], with possible variations in the definition itself (inanimate microorganisms, deliberately inactivated cells, etc.). Generally, postbiotics may be considered a complex mixture of metabolic products secreted by probiotics in cell-free supernatants (CFSs), such as enzymes, secreted proteins, short chain fatty acids, vitamins, secreted biosurfactants, amino acids, peptides, organic acids, etc., although purified components from microbial metabolism and vaccines are not considered postbiotics [4,5,6]. Postbiotics elicit several advantages over probiotics, including the following: (i) availability in their pure form, (ii) ease in production and storage, (iii) availability of production process for industrial-scale-up, (iv) specific mechanism of action, (v) better accessibility of Microbe-Associated Molecular Patterns (MAMPs) during the recognition of and interaction with Pattern Recognition Receptors (PRRs), and (vi) being more likely to trigger only the targeted responses by specific ligand–receptor interactions [6,7,8].

The outcome of a recent literature survey by Piqué et al. [9] underscores that postbiotics exert several pharmacodynamic features over live bacteria as follows: (i) no risk of bacterial translocation from the gut lumen to blood; (ii) no chance of the acquisition and transfer of antibiotic resistance genes; (iii) more natural to extract, standardize, transport, and store; (iv) further beneficial effects produced by a loss of viability due to cell lysis; and (v) an enhanced direct interaction between the released molecule from the disrupted cells and the epithelial cells [9,10].

Lactobacilli and *Bifidobacterium* spp. are among the most studied probiotic and/or functional genera, and the European Food Safety Authority (EFSA) has granted the Qualifed Presumption of Safety (QPS) status to them for human and animal applications, considering their safety perspectives [11,12,13].

Lactobacilli play a crucial role in the production of fermented foods, including vegetables, meats, and fermented dairy products. Over the last decade, the scientific understanding of lactobacilli (e.g., their metabolism and functions) has expanded considerably, opening the way to more reliable process control in production and in increasing the range of industrial applications as starters and adjunct starters/cultures (including probiotics) [14,15,16,17].

Bifidobacteria are considered a part of desirable microbiota, necessary for the proper functionality of the gastrointestinal tract. They colonize the gut and constitute the predominant microbiota of mucous membranes in the colon of breast milk-fed infants. In adult people, the bifidobacteria population varies depending on age, life condition, medical treatment, as well as eating habits [18,19].

When focusing on beneficial bacteria, there is a key factor to consider: the interplays and the connections among microorganisms belonging to different groups. In this context, the interplays between lactobacilli and bifidobacteria could play a significant role for their survival in complex matrices, like foods and gut microbiota. The interplays could be different and could include metabolic cooperation and cross-feeding, the production of stimulating molecules, and the release of signalling molecules able to promote an over-expression of some genes [20].

Amongst these different ways, the focus of this paper was on possible growth-promoting factors that are able to stimulate beneficial interplays between lactobacilli and bifidobacteria, generally known as the bifidogenic growth stimulator factor (BGS), firstly reported by Kaneko et al. [21] as a result of the metabolism of *Propionibacterium freudenreichii*.

The BGS is probably a mixture of 1,4-dihydroxy-2-napthoic acid (DNHA), 2-amino-3-carboxy-1,4-napthoquinone (ACQN), and other still unknown compounds [22,23], present in periplasm or in cytoplasm and released in cell-free extract. In this regard, BGS contained in CFSs might be considered as potential pre-postbiotic substances that are able to stimulate the growth/survival of the bifidobacteria of the intestinal microbiota [24], or nevertheless, it may be regarded as a parapostbiotic active mix.

The CFS produced by many probiotic microorganisms contains many bioactive compounds such as biosurfactants, bacteriocins, exopolysaccharide, active peptides, etc., and, to some extent, can be referred to, along with other soluble factors (products or metabolic byproducts) secreted by live bacteria, as postbiotic [25]. Some authors have in the past reported some evidence on CFSs produced by lactobacilli on bifidobacteria and vice versa [26]. However, to the best of the authors’ knowledge, it is not clear which effects of CFSs on these groups or which modulation of the growth/death kinetic was assessed, while the exact definition of the growth/survival profile of a probiotic microorganism in complex matrices is the main requisite to assess their effectiveness.

Thus, the main aim of this paper is to focus on the CFSs of some food-grade *Lactiplantibacillus plantarum* and *Bifidobacterium* strains as a way to promote positive interactions amongst these microorganisms, achieved by assessing the effects on the growth/death curve in a laboratory medium. A secondary goal was the shift from a qualitative to a quantitative evaluation of the effects, focusing on some fitting parameters that are able to measure the beneficial action in terms of survival and growth promotion. Finally, the strain influence was studied.

## 2. Materials and Methods

### 2.1. Microorganisms

*Lactiplantibacillus plantarum* c3, *L. plantarum* c4, and *L. plantarum* c15 were examined throughout this study for the bioactivity of their CFS towards bifidobacteria. All strains were isolated from Italian table olives “*Bella di Cerignola*” [27], identified and characterized [28], and stocked at −80 °C in appropriate media.

The bifidobacteria strains studied under the influence of *L. plantarum* CFS were the following: *Bifidobacterium animalis* DSM 10140, *Bifidobacterium subtile* DSM 20096, and *Bifidobacterium breve* DSM 20213, purchased from DSMZ (Deutsche Sammlung von Mikroorganismen und Zellkulturen GmbH, Germany), and stocked at −80 °C in appropriate media, as recommended by the producer (https://www.dsmz.de/).

Similarly, bifidobacteria were examined throughout this study for the bioactivity of their CFS towards lactobacilli.

### 2.2. Preparation of Cell-Free Supernatants

*L. plantarum* strains were grown in MRS broth (Oxoid, Milan, Italy) at 30 °C for 48 h, while *Bifidobacterium* spp. strains were grown in MRS broth (Oxoid, Milan, Italy) with cysteine (0.05% *w*/*v*) (cMRS) (Sigma-Aldrich, Milan, Italy) at 37 °C for 48 h. Each strain was grown separately.

After this step, the cells were harvested from the broths by centrifugation at 3000× *g* at 5 °C for 15 min and then washed twice with sterile saline solution (NaCl 9 g/L). The supernatants were filtered through a 0.22 µm Millipore filter (Whatman, Dassel, Germany), and the cell-free hydrosoluble fractions were recovered [21]. As a result, 6 different CFSs were produced, one for each strain, as follows: CFS_1_, from *L. plantarum* c3; CFS_2_, from *L. plantarum* c4; CFS_3_, from *L. plantarum* c15; CFS_4_, from *B. animalis* subsp. *lactis* DSM 10140; CFS_5_, from *B. subtile* DSM 20096; and CFS_6_, from *B. breve* DSM 20213.

### 2.3. Assays

Frozen cultures of *B. animalis*, *B. subtile*, and *B. breve* were thawed and pre-cultured in cMRS. The assays were performed in 250 mL Erlenmeyers, containing 100 mL of cMRS broth. The extracts produced from lactobacilli (CFS_1_, CFS_2_, CFS_3_) were separately added as a medium ingredient (1% *v*/*v*). The samples were inoculated with each strain of *Bifidobacterium* (10^2^ CFU/mL). Aliquots of cMRS, inoculated with the target strains but not containing CFSs, were used as controls. Thus, for each microorganism, 4 different experiments were carried out (control, medium + CFS_1_, CFS_2_, and CFS_3_). The samples were incubated under the optimal conditions of bifidobacteria (at 37 °C in anaerobic condition), and the viable count of bifidobacteria was periodically evaluated on cMRS agar, incubated at 37 °C for 48 h.

Frozen cultures of *L. plantarum* strains were thawed and pre-cultured in MRS broth and used to inoculate MRS (10^2^ CFU/mL), containing CFSs from bifidobacteria (CFS_4_, CFS_5_, CFS_6_) as an ingredient (1% *v*/*v*). MRS broth, inoculated with lactobacilli but not containing the extracts, was used as the control; each microorganism was analyzed individually assessing separately the effect of each CFS, thus focusing on 4 different batches for each strain (control, medium + CFS_4_, CFS_5_, and CFS_6_).

The samples were incubated at 37 °C (optimal temperature for lactobacilli, at least for the functional traits; 28) in anaerobic conditions, and the viable count of *L. plantarum* was periodically evaluated on MRS agar, incubated at 30 °C for 48 h.

### 2.4. Modelling

The viable count of each strain, with and without CFSs, was modelled with two different functions: a positive Gompertz equation for the growth part (lag phase, exponential, and steady state) and a negative Gompertz equation (for this function, the growth part of the kinetic was assumed as a shoulder length, followed by a liner death kinetic and finally by a tail).

For the growth part, the Gompertz equation reparameterized by Corbo et al. [29] was used as follows:

Log (CFU) = [log(CFU)]_max_ − A × exp{−exp{[(µ_max_ × 2.7182) × (λ − t-7log)/A] + 1}}

+ A × exp{−exp{[(µ_max_ × 2.7182) × (λ − t)/A] + 1}}

where [log(CFU)]_max_ is the standard limit for the microbial population (7 log CFU/mL), A is the maximum increase in the bacterial load attained at stationary phase, $\mu_{\max}$ is the maximal growth rate [Δlog (CFU/mL)/h], λ is the lag time (hours), and t* is the t-7log; that is, the verge time (hours) after which the cell load attained a value higher than 10^7^ CFU/mL.

For the negative part of the growth curve, the reparameterized Gompertz equation was cast as follows:

Log (CFU) = [log(CFU)]_max_ − {−Δ × exp{−exp{[(d_max_ × 2.7182) × (λ − t-7log*)/Δ] + 1}}

+Δ × exp{−exp{[(d_max_ × 2.7182) × (α − t)/Δ] + 1}}}

where [log(CFU)]_max_ is the standard limit for the microbial population (7 log CFU/mL), Δ is the maximum decrease in the bacterial load attained in the death phase, $d_{\max}$ is the maximal death rate [Δlog (CFU/mL)/h], α is the shoulder length, after which the decrease in cell load started (hours), t is the time, and t-7log* is the time limit, after which the cell load attained a value lower than 10^7^ CFU/mL (h).

The difference between t-7log* and t-7log was defined as the stability time; that is, the time when the target strain addressed the basic requisite of a probiotic microorganism in a matrix (viable count at least 7 log CFU/mL). The reparameterized Gompertz equation was originally developed for the mathematical definition of the shelf life (intended as the time before a critical threshold is attained); its main difference, compared to the classical approach for shelf-life definition, is that it allows for the evaluation of the variability of shelf life itself, as it is a primary fitting parameter for the model and it is not calculated as a ratio or through an indirect formula. In this research, the shelf-life parameter was used to evaluate t-7log and t7-log*, along with a robust measure of their variability.

In addition, considering the classical Gompertz equation, reparameterized by Zwietering et al. [30], the model has a sigmoidal shape and allows for the estimation of all the phases of a growth kinetic; moreover, it is simple in its linear form, and a simple sign change could be used to model both growth and death kinetics, while this approach is quite difficult for other primary functions (for example, Baranyi or logistic models).

An indirect parameter was then evaluated as the difference between t-7log* and t-7log as it provides a measure of the time when the concentration of the target strain was >7 log CFU/mL (stability time).

After the primary modelling, a secondary modelling approach was tested through the multifactorial analysis of variance (ANOVA) for 3 parameters (t-7log, t-7log*, and stability time) to analyze the effect of two independent variables; that is, the kind of microorganisms (the target strain of lactobacilli or bifidobacteria) and the kind of extracts (CFS_1_, CFS_2_, CFS_3_ for the first experiment or CFS_4_, CFS_5_, and CFS_6_ for the second experiment).

However, an issue to face initially was the variability connected to the growth/death kinetic of each strain, which could mask the statistic effect of the two predictors; thus, the control was used as a baseline to standardize t-7log, t-7log*, and the stability time, which were then expressed as an increase or decrease in the control. After this standardization step, the parameters were analyzed. ANOVA was performed first in the classical form of evaluating the variability within each group and the variability among groups; then, a post hoc and multiple comparison approach was carried out through Tukey’s test (*p* < 0.05).

Statistics were performed through the software Statistica for Windows, ver. 7.0 (Statsoft, Tulsa, OK, USA).

## 3. Results

The viable count of the target strains (*L. plantarum* in the presence of CFSs from bifidobacteria and *Bifidobacterium* spp. in the presence of CFSs from *L. plantarum*) were fitted through the Gompertz equation, cast both in the positive form for the first part of the death kinetic (from lag phase to the steady state) and the negative form for the second part (steady state or shoulder phase, death kinetic), as a complete model with all the phases was used in a preliminary phase and led to an overestimation of some fitting parameters, mainly on the growth and death rates. The choice of the target strains relied upon the promising results found in the preliminary phase when the extracts of propionibacteria had been added to laboratory media [24]. Moreover, the technological and functional properties of the strains used in this research are well known, as are their growth kinetic under optimal conditions.

For the aim of this research, two parameters were further studied and analyzed for both *L. plantarum* and *Bifidobacterium* spp.; that is, the t-7log (the time to attain a 7 log CFU/mL viable count in the exponential phase) and t-7-log* (the time after which the viable count was reduced below 7 log CFU/mL in the death phase). The difference between t-7log* and t-7 log defined the stability time (ST); that is, the time when the viable count of the target strains was >7 log CFU/mL.

The analysis of the fitting parameters revealed that the addition of a CFS determined a reduction in the t-7log of bifidobacteria and *L. plantarum* due to a stimulation in the exponential phase, along with an increase in the stability time, due to a possible protective effect.

An issue when analyzing data of different species or strains is that they could not be simply compared, as they are referred to as different biological systems; therefore, a preliminary standardization to delete the “biological variability” is necessary.

In this paper, the standardization was achieved by reporting the data concerning decreases in t-7log (difference between t-7log in the control and t-7 log in the samples with CFSs) to point out the stimulation and increases in the stability time to point out the protective effect.

These new standardized parameters were used as dependent variables for a two-way ANOVA, where the categorical predictors were the target strains and the kind of CFS.

The first output of ANOVA was a table of standardized effects (Table 1), which shows the statistical weight of the individual terms of predictors as well as of their interaction.

The t-7log reduction in *Bifidobacterium*, when the CFS from *L. plantarum* was used, was affected by the individual terms, although the most significant was the kind of CFS. On the other hand, t-7 log reduction in *L. plantarum* was influenced by the individual term “microorganism” (kind of target strain) and secondarily by the interactive term.

Finally, the ST increase in *L. plantarum* was significantly linked to the predictors (kind of CFS, microorganisms, and interactive term).

A table of standardized effects cannot offer quantitative results, but only qualitative (if a predictor is significant or not). Quantitative outputs can be gained through the decomposition of the statistical hypothesis, which does not show an actual trend. It is a mathematical function showing the contribution of each variable to the dataset.

**Figure 1:**
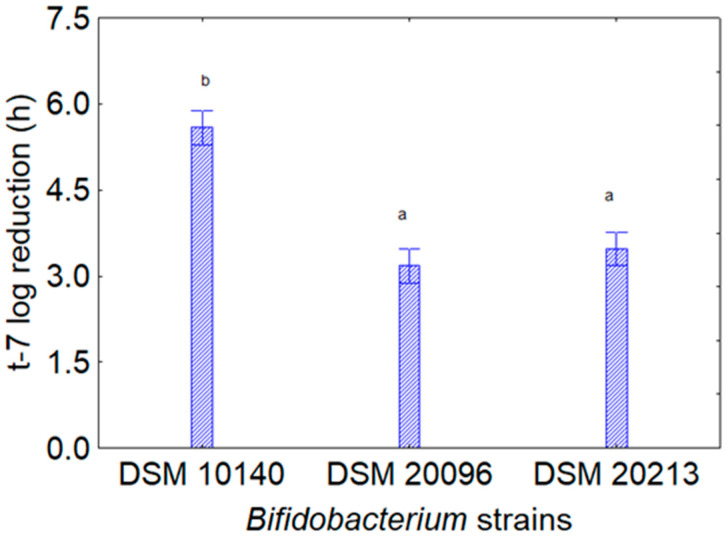
Decomposition of the statistical hypothesis for the individual effect of the predictor “target microorganism” on the assay *Bifidobacterium* spp. and CFS of *L. plantarum* on the reduction in t-7 log (that is, the reduction in time to attain a viable count of 7 log CFU/mL by the target strain). Bars denote 95% confidence interval, while letters indicate significant differences (Tukey’s test, *p* < 0.05). The picture shows the cumulative results for the aforementioned extracts.

Figure 1 shows the contribution of the kind of microorganisms for the assay *Bifidobacterium* +CFS from *L. plantarum* (CFS_1_, CFS_2_ or CFS_3_); as reported above, the addition of CFSs generally had a positive and stimulating effect with a decrease in t7-log. The strain most affected was *B. animalis* subsp. *lactis* DSM 10140, which experienced a reduction of 5.8 h, while for the other two strains, t-7 log was reduced by ca. 3 h.

**Figure 2:**
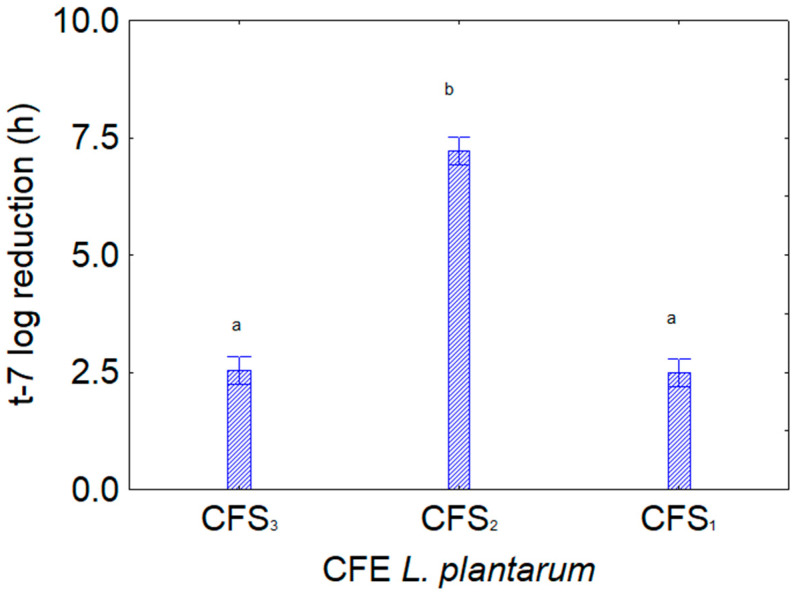
Decomposition of the statistical hypothesis for the individual effect of the predictor “kind of CFS” on the assay *Bifidobacterium* spp. and CFS of *L. plantarum* (CFS_1_, *L. plantarum* c3; CFS_2_, *L. plantarum* c4; CFS_3_, *L. plantarum* c15) on the reduction in t-7 log (that is, the reduction in the time taken to attain a viable count of 7 log CFU/mL by the target strain). Bars denote 95% confidence interval, while letters indicate significant differences (Tukey’s test, *p* < 0.05). This figure shows the cumulative effects of the different extracts on all bifidobacteria.

For the same experiment, the contribution of the predictor CFS is in Figure 2. The most effective extract was CFS_2_ (from *L. plantarum* c4), which caused a t-7log reduction of ca. 7.2 h.

**Figure 3:**
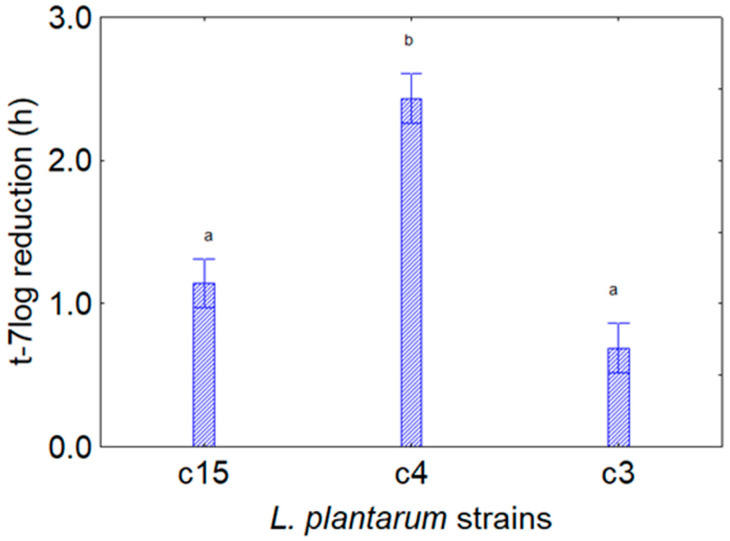
Decomposition of the statistical hypothesis for the individual effect of the predictor “target microorganism” on the assay *L. plantarum* and CFS of *Bifidobacterium* spp. on the reduction in t-7 log (that is, the reduction in the time taken to attain a viable count of 7 log CFU/mL by the target strain). Bars denote 95% confidence interval, while letters indicate significant differences (Tukey’s test, *p* < 0.05). The picture shows the cumulative results for the aforementioned extracts.

For the second experiment (*L. plantarum* + CFS from *Bifidobacterium*), Figure 3 shows the contribution of the predictor microorganism, with a slight effect on the strains c3 and c15 (mean reduction in t-7log by 1 h) and a higher effect on the strain c4 (reduction of 2.5 h), thus suggesting a general positive effect of CFS_4_, CFS_5_, and CFS_6,_ independently from the kind of extract.

**Figure 4:**
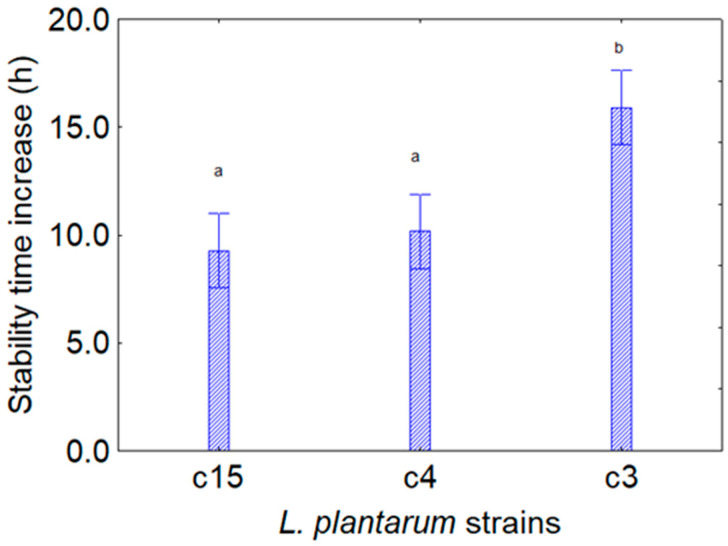
Decomposition of the statistical hypothesis for the individual effect of the predictor “target microorganism” on the assay *L. plantarum* and CFS of *Bifidobacterium* spp. on the increase in the stability time (that is, the time when the viable count of the target strain was at least 7 log CFU/mL). Bars denote 95% confidence interval, while letters indicate significant differences (Tukey’s test, *p* < 0.05). The picture shows the cumulative results for the aforementioned extracts.

For this second experiment, the most important effect was on the stability time, as it was strongly increased in the presence of CFSs from *Bifidobacterium*, with a mean increase of 9 h for the strains c15 and c4 and 15 h for the strain c3 (Figure 4), independently from the kind of extracts.

**Figure 5:**
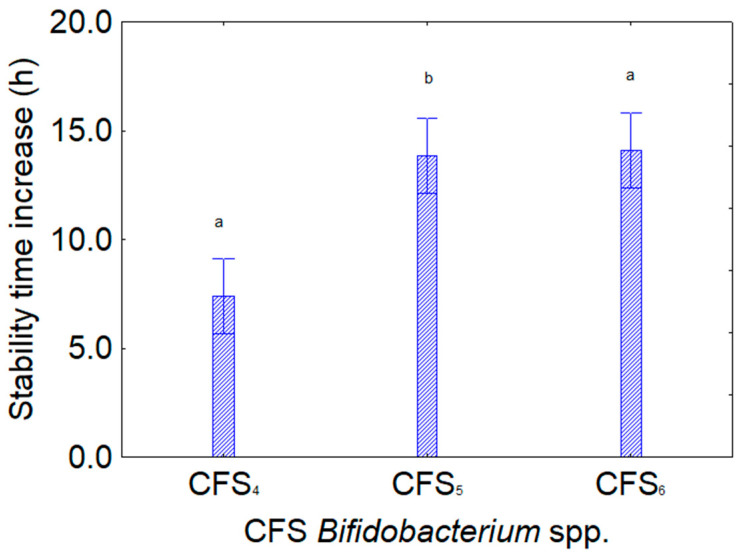
Decomposition of the statistical hypothesis for the individual effect of the predictor “kind of CFS” on the assay *L. plantarum* and CFE of *Bifidobacterium* spp. (CFS_4_, *B. animalis* subsp. *lactis*; CFS_5_, *B. subtile* DSM 20096; CFS_6_, *B. breve* DSM 20213) on the increase in the stability time (that is, the time when the viable count of the target strain was at least 7 log CFU/mL). Bars denote 95% confidence interval, while letters indicate significant differences (Tukey’s test, *p* < 0.05). The figure shows the cumulative effects of the different extracts on all bifidobacteria.

**Figure 6:**
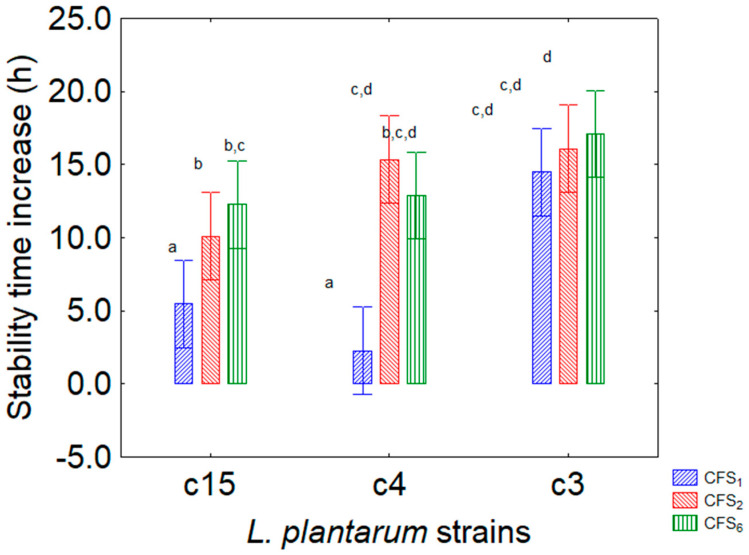
Decomposition of the statistical hypothesis for the interactive effect “kind of CFS × target strain” on the assay *L. plantarum* and CFS of *Bifidobacterium* spp. on the increase in the stability time (that is, the time when the viable count of the target strain was at least 7 log CFU/mL). Bars denote 95% confidence interval, while letters indicate significant differences (Tukey’s test, *p* < 0.05). CFS_4_, *B. animalis* subsp. *lactis*; CFS_5_, *B. subtile* DSM 20096; CFS_6_, *B. breve* DSM 20213.

Figure 5 shows the contribution of the kind of extracts from bifidobacteria, with a higher effect for the CFS_5_ and CFS_6_ (14 h, from *B. subtile* and *B. breve*) and a lower effect for *B. animalis* subsp. *lactis* (CFS_4_, 7.5 h). Finally, Figure 6 shows the importance of the interaction “kind of microorganism” × “kind of CFS”, as it highlights that some combinations were not or less effective (strains c4 and c15 with CFS_4_ from *B. animalis*), while others had generally higher performances (for example, those where CFS_5_ and CFS_6_, from *B. subtile* and *B. breve*, were added).

## 4. Discussion

Gut microbiota composition can play an important role in the host’s health status, and microbiota disruption has been linked to diarrhea [31], irritable bowel syndrome (IBS) [32], obesity [33,34], allergies [35], behavioural and developmental disorders, including autism [36], and neurological diseases [37]. Altered levels of microbial metabolites have also been associated with many conditions, including depression [38], colorectal cancer, cardiovascular disease, obesity, and type 2 diabetes [39]. Strategies designed to influence microbiota composition include the ingestion of probiotics, prebiotics, and synbiotics (a combination of selected substrates and live bacteria that provide a health benefit) [40].

In some cases, the direct use of probiotics can be dangerous for some people; for example, those with reduced immunity, an impaired intestinal barrier, sepsis, and premature babies [41,42]. Hence, the concept of administering inactivated bacterial strains, bacterial fragments, and products of their metabolism to obtain similar effects is highly useful [25].

Metabolites/CFSs and soluble factors (products or metabolic byproducts) secreted by living bacteria may be considered postbiotics, also referred to as metabiotics; that is, components or metabolites produced by probiotics able to exert positive effects on host functions and microbiota [43]. This is a novel approach in food microbiology, as in the past, CFS preparations were extensively studied as antimicrobial preparations towards a wide variety of pathogens [44,45,46,47], but their use as promoting factors or as ingredients to gain positive effects in the gut microbiota was not fully explored at least to the authors’ knowledge.

The present study demonstrated a possible mutual protective and/or stimulating effect between the tested bacteria and a species/specific variability. In our conditions, *L. plantarum* c4 showed the best performances, while the differences among bifidobacteria were less pronounced, and further investigations could explain the specific mechanisms of action able to enhance the probiotic potentials by using a parapostbiotic approach in microbial applications in food and pharma.

It is well known that various bifidobacterial species with complementary metabolic abilities have been reported to cooperate in the use of complex carbohydrates in vitro [48] and to enhance the in vivo persistence levels of each strain when they co-occur, and, in addition to such a mutualism within the genus, bifidobacterial strains can expand the availability of carbohydrates for other gut symbionts, i.e., members of Bacteroidetes [49].

A positive effect was also recorded for CFSs produced by lactobacilli, thus also suggesting that the species *L. plantarum* could produce a bifidogenic factor with a promoting effect, as also evidenced by Tan et al. [50] for the interaction CFS from *L. plantarum* and *Enterococcus faecium*. The nature of these compounds is not known, but some evidence suggests the presence of the supernatant of amino acids, including glutamic acid and proline [43]; in addition, lactobacilli and bifidobacteria can produce short chain fatty acids (SCFAs), which are known to promote growth, or can release peptides (as endopeptidases) and/or cationic species into the medium, which could then enter cells or interact with the external layers [51,52].

Amino acids and SCFAs, along with other unknown complex metabolites, could be responsible for the stimulation evidenced in the growth phase for both lactobacilli and bifidobacteria, as highlighted by the reduction in t-7log. In addition, another positive effect found in this paper is the protective effect in the steady state and senescence, as pointed out by the increase in the stability time. Some data from the literature suggest that this effect could be linked to antioxidant activity, counteracting the action of free radicals, and probably due to the presence of pyruvic, linolenic, lactic, and indole lactic acids [53].

More strong evidence emerging from the results of this paper concerns the potentiality of *L. plantarum* and bifidobacteria, but also the strong strain-dependence of the positive effects, linked to both the strain-producing CFS and the target strain, thus stressing the importance of the correct combination for good mutualistic growth enhancement or protective effects in the death phase.

From a practical point of view, the results could provide a background for the design of new synbiotic strategies (combination of prebiotics and probiotics), where CFSs act as prebiotic compounds that are able to prolong the survival of probiotic microorganisms in food matrices. In particular, the use of the reparameterized Gompertz equation allowed for a quantitative estimation of the beneficial effect in terms of the stability time. This approach could be used in the future to define combinations of CFSs and probiotics for functional foods with a prolonged shelf life. However, the experiments of this research were conducted at 37 °C, while it is safe to assume that the effects could have been enhanced under refrigeration or at lower temperatures; that is, under the real storage conditions of probiotic foods. Thus, an effective validation in foods and under the real storage conditions is required.

Another possibility is the design of a new combination of functional strains able to stimulate each other through release into the medium of various metabolites, combining a multi-strain cocktail and the presence of stimulating ingredients that are able to promote the functionality of probiotics. However, the strain-specific effects highlight the importance of personalized combinations of strains/species as the cross-feeding of probiotic bacteria represents a valuable strategy both for food and for the restoration of a eubiotic status in the gut microbiota [20], but it is necessary to highlight the exact mode of action of these interactions and of the differences amongst the species of a genus (as in the case of *Bifidobacterium* spp.) or of the strains inside the species (as for *L. plantarum*). Future research in this context could be focused on the definition of the metabolic pathways beyond the positive effects of CFSs.

## 5. Conclusions

This research contributed to the ongoing debate on the mutualistic interactions and interplays between lactobacilli and bifidobacteria, using a simple laboratory medium as the substrate and containing the extracts produced by the microorganisms as active ingredients. The use of the reparameterized Gompertz equation and the evaluation of the growth and death kinetic highlighted a general positive effect during both the growth and death steps, with an increase in the stability time for all lactobacilli and bifidobacteria, thus suggesting the potentialities of extracts as cross-feeding approaches for probiotic microorganisms. However, an important point was the strong strain dependence, concerning both the target strain and the microorganism producing the extract, with some bifidobacteria (*B. breve* and *B. subtile*) producing CFSs more effective than others or some *L. plantarum* (e.g., strain c4) being more affected by the presence of active ingredients.

The study was conducted under strictly controlled conditions and in a simple medium; thus, a confirmation step on a wide variety of strains, complex matrices, and also under the conditions that mimic the gut are necessary, as well clarifying the mechanisms beyond the positive effects.

## Figures and Tables

**Table 1:** Individual standardized and interactive effects of microorganisms, kind of CFS on t7-log reduction (that is, the reduction in the time taken to attain a viable count of 7 log CFU/mL by the target strain), and the stability time increase (ST; that is, the time when the viable count of the target strain was at least 7 log CFU/mL).

	SS *	Df	MS	F-Test
t-log reduction, *Bifidobacterium* ssp. and CFS from *L. plantarum*				
Microorganism	31.09	2	15.54	85.90
CFS	133.49	2	66.75	368.87
Interaction	/**	/	/	/
t-7 log reduction, *L. plantarum* and CFS from *Bifidobacterium* spp.				
Microorganism	14.73	2	7.36323	122.99
CFS	/	/	/	/
Interaction	1.83	4	0.46	7.67
ST increase, *L. plantarum* and CFS from *Bifidobacterium* spp.				
Microorganism	232.25	2	116.13	19.18
CFS	258.20	2	129.10	21.32
Interaction	114.93	4	28.73	4.75

* SS, sum of square; df, degree of freedom; MS, mean residual square; F-test, Fisher test. **, not significant.

## Data Availability

The original contributions presented in the study are included in the article, further inquiries can be directed to the corresponding author.
